# Viral Regulation of RNA Granules in Infected Cells

**DOI:** 10.1007/s12250-019-00122-3

**Published:** 2019-04-29

**Authors:** Qiang Zhang, Nishi R. Sharma, Zhi-Ming Zheng, Mingzhou Chen

**Affiliations:** 1 State Key Laboratory of Virology and Modern Virology Research Center, College of Life Sciences, Wuhan University, Wuhan, 430072 China; 2 Tumor Virus RNA Biology Section, RNA Biology Laboratory, National Cancer Institute, National Institutes of Health, Frederick, MD 21702 USA

**Keywords:** Stress granules (SG), P-bodies (PB), RNA virus, DNA virus

## Abstract

RNA granules are cytoplasmic, microscopically visible, non-membrane ribo-nucleoprotein structures and are important posttranscriptional regulators in gene expression by controlling RNA translation and stability. TIA/G3BP/PABP-specific stress granules (SG) and GW182/DCP-specific RNA processing bodies (PB) are two major distinguishable RNA granules in somatic cells and contain various ribosomal subunits, translation factors, scaffold proteins, RNA-binding proteins, RNA decay enzymes and helicases to exclude mRNAs from the cellular active translational pool. Although SG formation is inducible due to cellular stress, PB exist physiologically in every cell. Both RNA granules are important components of the host antiviral defense. Virus infection imposes stress on host cells and thus induces SG formation. However, both RNA and DNA viruses must confront the hostile environment of host innate immunity and apply various strategies to block the formation of SG and PB for their effective infection and multiplication. This review summarizes the current research development in the field and the mechanisms of how individual viruses suppress the formation of host SG and PB for virus production.

## Introduction

While the intracellular environment and embedded cellular machinery provide the needed vital force and necessary materials for viruses to replicate after infection, these host machineries are not available to these foreign invaders at ease. In fact, viruses have to counter the multiple layers of intracellular defense to replicate and establish their dominance for their propagation. RNA granules (Thomas *et al.*^2011^) are dynamic non-membrane subcellular structures (Ivanov *et al.*^2018^) containing translationally silenced messenger ribonucleoproteins (mRNPs), which play an important role in regulation of cellular homeostasis, RNA metabolism and gene expression at the posttranscriptional level (Anderson and Kedersha 2009). Stress granules (SG) and processing bodies (PB) (Eulalio *et al.*^2007^) are two of RNA granules well characterized in yeast and mammalian cells (Poblete-Duran *et al.*^2016^) and are important components of the host cell antiviral defense.

**Fig. 1:**
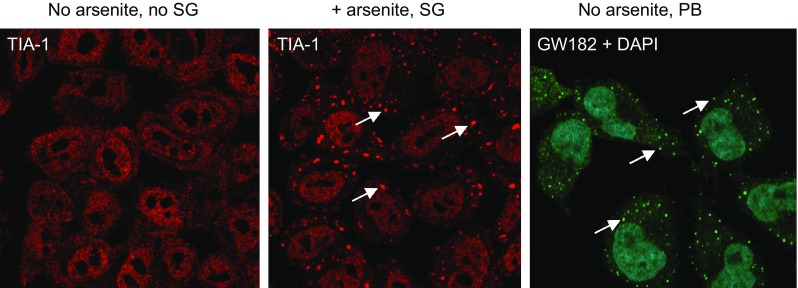
Mammalian RNA granules. HeLa cells immunostaining with anti-TIA-1 (left and middle, red) show stress granules (SG) during stress of NaAS_2_O_3_ (+arsenite, middle) and with anti-GW182 show processing bodies (PB) under physiological condition. Arrows indicate granules (SG or PB).

SG are non-membranous, transiently assembled cytoplasmic aggregates of 48S mRNPs and associated proteins (Stohr *et al.*^2006^; Buchan and Parker 2009), where stalled translation preinitiation complexes (PICs) repress the translation of nonessential mRNAs (Anderson *et al.*^2015^) and modulate cell signaling by sequestering key signal translation proteins (Kedersha *et al.*^2013^). Thus, SG are thought to be the aggregates of stable, translationally silent mRNAs (Kedersha and Anderson 2002). A variety of environmental stresses, including viral infection, can trigger SG formation in eukaryotic cells (Anderson and Kedersha 2008). In contrast, PB can exist in the absence of stress (Stoecklin and Kedersha 2013), which are sites of active mRNA decay (Decker and Parker 2012). SG initiate global translational arrest by storing mRNA (Anderson and Kedersha 2009) for exchange with either polysomes for translation or PB for degradation (Kedersha *et al.*^2005^). RNA-binding proteins TIA-1 (Kedersha *et al.*^1999^; Gilks *et al.*^2004^), G3BP (Tourriere *et al.*^2003^; Matsuki *et al.*^2013^) and PABP (Ma *et al.*^2009^; Smith and Gray 2010; Burgess *et al.*^2011^) are three fundamental components of SG during stress (Fig. 1). GW182 and de-capping/de-adenylating enzymes are specific components of PB (Kedersha *et al.*^2005^), where siRNA- or miRNA-guided mRNAs are processed and degraded (Liu *et al.*^2005^) (Fig. 1). Virus infection imposes stress on host cells (McInerney *et al.*^2005^) and thereby induces SG formation. SG can shut off the translation of bulk mRNAs (Poblete-Duran *et al.*^2016^) to regulate gene expression and compartmentalization of heterologous viral RNAs and proteins. At the same time, viruses must take strategies to confront these responses and maximize their own replication efficiency (White and Lloyd 2012) by inhibition of SG formation and disruption of PB assembly via virally encoded factors.

## Viral Regulation of RNA Stress Granule Formation

### SG Formation and Induction of SG by RNA Virus Infections

**Fig. 2:**
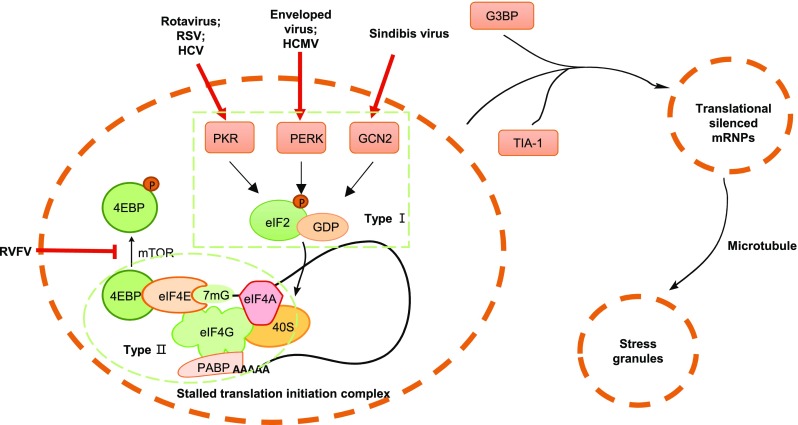
Viruses induce SG formation. Type I SG formation: RNAs derived from rotavirus, RSV and HCV activate PKR; High levels of glycoproteins produced from enveloped virus activate PERK; HCMV infection activates PERK; Sindbis virus genomic RNA activates GCN2. Type II SG formation: RVFV attenuates mTOR signaling to inhibite 4EBP phosphorylation. All above lead to the formation of stalled translation complexes to initiate the assembly of SG.

The process of SG formation can be artificially divided into the following steps (Fig. 2): (1) accumulation of stalled translation initiation complexes (Panas *et al.*^2016^) in response to various types of stress; (2) the RNA-binding proteins such as RAS-GTPase-activating protein SH3 domain-binding protein 1 (G3BP1) and T cell-restricted intracellular antigen 1 (TIA1) bind mRNAs and aggregate to nucleate SG formation. Self-aggregation of G3BP1 (Tourriere *et al.*^2003^) and the binding of TIA1 and TIAR (TIA-1-related protein) to polysome-free mRNAs, which exposes prion-like domains (Gilks et al. 2004), trigger mRNP aggregation. The aggregation of proteins is dynamic, and can rapidly exchange between SG and cytosol (Kedersha *et al.*^2000^, ^2005^). (3) large SG aggregate from smaller foci via posttranslational modification and microtubule transport (McCormick and Khaperskyy 2017). Many SG proteins undergo multiple post-translational modifications (Jayabalan *et al.*^2016^; Protter and Parker 2016). For example, G3BP1 must be demethylated (Tsai *et al.*^2016^), dephosphorylated (Kedersha *et al.*^2016^) and poly(ADP)-ribosylated (Leung *et al.*^2011^) to promote SG nucleation. Accordingly, SG formation also requires ongoing transport of mRNPs along with an intact microtubule cytoskeleton (Ivanov *et al.*^2003^). Theoretically, viral interference with any of these important steps may modulate SG formation in cells. In fact, many viral factors can interfere with SG formation and/or function. Meanwhile, SG can entrap viral RNA in some cases (McCormick and Khaperskyy 2017). Therefore, SG are thought to be antiviral (Rozelle *et al.*^2014^). Thus, to illustrate the relationship between SG and RNA viruses would be important for us to better understand the interactions of host and viruses.

Up to the present, SG can be divided into two types according to their formation mode. Type I SG formation depends on phosphorylation of eukaryotic translation initiation factor-2α (eIF2α) by one of the eIF2 kinases—double-stranded RNA (dsRNA)-activated protein kinase or protein kinase R (PKR) (Srivastava *et al.*^1998^; Garcia *et al.*^2007^; Onomoto *et al.*^2012^), PKR-like ER kinase (PERK) (Harding *et al.*2000a, b), general control non-derepressible protein 2 (GCN2) (Wek *et al.*^1995^; Deng *et al.*^2002^) or haeme-regulated inhibitor (HRI) (McEwen *et al.*^2005^), which are activated by distinct types of stress. Phosphorylated eIF2α stably binds to eIF2β, which prevents the recycle of eIF2 and regeneration of the eIF2-GTP-Met-tRNA_i_^Met^ ternary complex. Thus, eIF2α phosphorylation blocks recognition of the initiation codon and joining of the large ribosomal subunit, resulting in accumulation of stalled 48S mRNPs (Jackson *et al.*^2010^). Type II SG formation is independent of eIF2α phosphorylation, but requires eIF4F complex disruption such as inhibition of eIF4A RNA helicase (Bordeleau *et al.*^2006^; Dang *et al.*^2006^) or disruption of eIF4E activity (von der Haar *et al.*^2004^; Fournier *et al.*^2013^) for recognition and binding of RNA cap structure. The stress induced by nutrient, energy, oxygen or growth factor insufficiency inhibits mTOR complex 1 (mTORC1), whose activity is required for the dissociation of 4EBPs from eIF4E (Fujimura *et al.*^2012^) and enables eIF4E to form the eIF4F complex, and thus blocks assembly of pre-initiation complexes (Zoncu *et al.*^2011^).

**Fig. 3:**
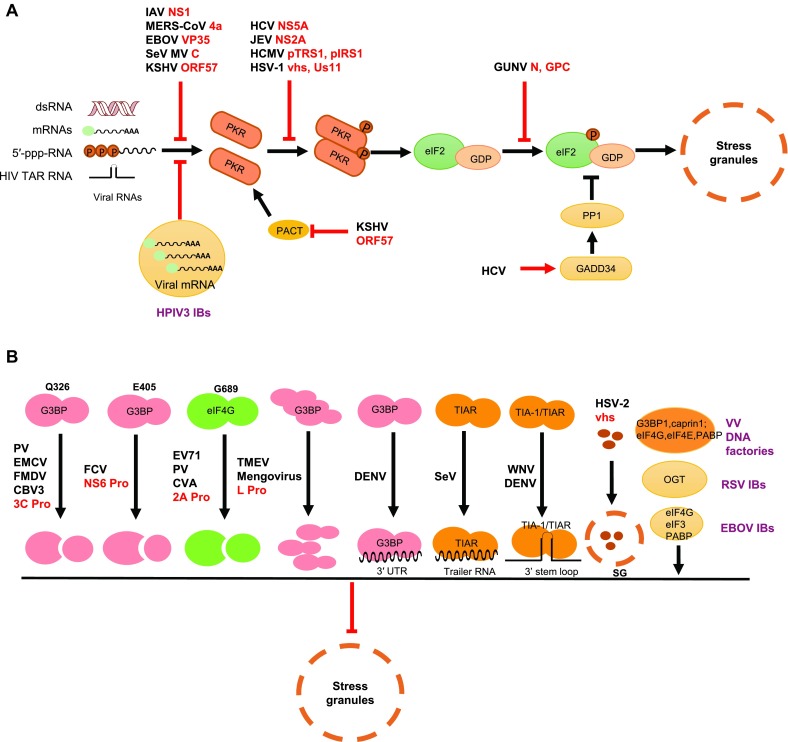
Viruses interfer with SG formation. **A** Viruses modulate eIF2α phosphorylation. IBs of HPIV3 shield viral RNAs from recognition by PKR; IAV NS1, MERS-CoV accessory protein 4a, EBOV VP35, SeV and MV C protein and KSHV ORF57 prevent viral dsRNA from binding by PKR; ORF57 interacts with PACT to prevent PKR activation; HCMV pTRS1 and pIRS1 and HSV-1 vhs and Us11 block PKR activation; HCV NS5A and JEV NS2A interact with PKR and prevent PKR dimerization; N and GPC of JUNV impair the phosphorylation of eIF2α; HCV modulates GADD34 and PP1 to de-phosphorylate eIF2α. **B** Viruses modulate SG formation downstream of eIF2α phosphorylation. 3C protease of PV, EMCV, FMDV and CVB3 cleaves G3BP at Q326; FCV NS6 protein cleaves G3BP at E405; 2A protease of EV71, PV and CVA cleaves eIF4G at G689; L protein of both TMEV and mengovirus inhibits G3BP1 aggregation; DENV 3′-UTR interacts with G3BP; SeV Trailer RNA captures TIAR from SG; WNV and Dengue virus (DENV) 3′-end genome captures TIA-1/TIAR; HSV-2 vhs localizes to SG and its endoribonuclease activity is required to disrupt SG formation; EBOV and RSV sequester SG proteins within viral inclusion bodies; VV sequesters crucial SG components within DNA factories.

Type I SG formation induced by viruses is the most and best-studied example (Table 1, Fig. 3A). Various RNA products derived by viruses including long dsRNA (Rojas *et al.*^2010^), 5′-triphosphate RNA (5′-ppp-RNA) (Nallagatla *et al.*^2007^), dsRNA that is formed by the antiparallel mRNA transcripts of some DNA viruses (Willis *et al.*^2011^), and human immunodeficiency virus (HIV) transactivation-response region (TAR) RNA hairpins (Heinicke *et al.*^2009^), can be recognized by PKR. The activated PKR initiates SG assembly through eIF2α phosphorylation. For instance, the persistent phosphorylation of eIF2α (Montero *et al.*^2008^) during rotavirus infection is PKR-dependent as a consequence of the accumulation of viral dsRNA in the cytoplasm outside the viroplasms (virus-induced cytoplasmic inclusion bodies called viroplasms [VMs]) (Rojas *et al.*^2010^). Even though eIF2α is phosphorylated in rotavirus-infected cells, the formation of SG is prevented and viral proteins are efficiently translated, suggesting that the virus prevents the assembly of these structures presumably downstream of eIF2α phosphorylation to allow the translation of its mRNAs (Mazroui *et al.*^2006^). Very recently, Dhillon and Rao found that rotavirus induces formation and sequestration of remodeled SG and PB in the VMs which contain the majorities of their components but selective exclusion of a few proteins (G3BP1 and ZBP1 for SG, DDX6, EDC4 and Pan3 for PB), to promote virus replication (Dhillon and Rao 2018). Oceguera *et al.* demonstrated that viral RNA of rotavirus could interact with several RNA binding proteins (RBPs) (Xrn1, Dcp1, Ago2, Hur) and interfere with their subcellular localization (Oceguera *et al.*^2018^). Lindquist *et al.* (Lindquist *et al.*^2011^) first determined that SG induction by respiratory syncytial virus (RSV) was mediated by PKR-dependent eIF2α phosphorylation. The RSV-mediated SG formation was significantly reduced in PKR-knockdown cells (Lindquist *et al.*^2010^). In addition, it has been shown that Hepatitis C virus (HCV) strongly activates PKR via the 5′-untranslated region (UTR) of its genome (Toroney *et al.*^2010^), thereby inducing SG. NS1-mutant Influenza virus A (IAV) (Khaperskyy *et al.*^2012^; Mok *et al.*^2012^; Ng *et al.*^2013^) and C protein-deficient Sendai virus (SeV) (Takeuchi *et al.*^2008^) lead to significant activation of PKR and eIF2α phosphorylation. Besides, PERK could be activated by high levels of glycoproteins produced from enveloped viruses (Chan and Egan 2005), and general control non-derepressible-2 (GCN2) could be activated by Sindbis virus (SINV) genomic RNA (Berlanga *et al.*^2006^), both leading to phosphorylation of eIF2α. GCN2 prevents replication of SINV in the early stages of the viral replicative cycle by blocking the synthesis of NSPs from SINV RNA (Berlanga *et al.*^2006^; Frolova *et al.*^2006^; Gorchakov *et al.*^2008^).

**Table 1:** Regulation of SG by viruses.

Genome	Virus family	Virus	Type	Mechanism	References
dsDNA	*Herpesviridae*	HCMV	Induction	Modifies the UPR and activates PERK	Isler *et al*. (2005)
			Inhibition	pTRS1 and pIRS1 antagonize PKR to facilitate virus replication	Ziehr *et al.* (2016)
		KSHV	Inhibition	ORF57 interacts with PKR and PACT to inhibit PKR activation	Sharma *et al*. (2017)
		HSV-1	Inhibition	vhs and Us11 protein play a key role in blocking the activation of PKR	Sciortino *et al*. (2013)
		HSV-2	Inhibition	vhs localizes to SG and its endoribonuclease activity is required to disrupt SG formation	Finnen *et al*. (2012, 2014, 2016)
	*Poxviridae*	VV	Inhibition	Sequesters crucial SG components within DNA factories	Katsafanas and Moss (2007), Zaborowska *et al*. (2012)
			Induction	Untranslated mRNA accumulation in viral DNA factories induces RNA granules formation	Meng and Xiang (2019)
dsRNA	*Reoviridae*	Rotavirus	Induction	Phosphorylation of eIF2α is PKR-dependent as a consequence of the accumulation of viral dsRNA	Montero *et al*. (2008), Rojas *et al*. (2010)
			Modulation	Induces formation and sequestration in the VMs of remodeled SG and PB	Dhillon and Rao (2018)
(+)ssRNA	*Picornaviridae*	PV	Inhibition	Viral 3C protease cleaves G3BP	White *et al*. (2007)
		FMDV	Inhibition	Viral 3C protease cleaves G3BP	Ye *et al*. (2018)
				Leader Protease Cleaves G3BP1 and G3BP2	Visser *et al*. (2019)
		TMEV	Inhibition	Express the leader (L) protein to inhibit G3BP1 aggregation	Borghese and Michiels (2011)
		Mengovirus	Inhibition	Express the leader (L) protein to inhibit G3BP1 aggregation	Borghese and Michiels (2011)
		EV71	Modulation	2A protease inhibits typical SG formation but induces atypical SG formation by cleaving eIF4GI	Yang *et al*. (2018)
	*Caliciviridae*	FCV	Inhibition	NS6Pro cleaves G3BP1	Humoud *et al*. (2016)
	*Togaviridae*	SINV	Induction	Genomic RNA activates GCN2	Berlanga *et al*. (2006)
	*Flaviviridae*	WNV	Inhibition	3′-end viral genome captures TIA-1/TIAR	Li *et al*. (2002, Emara and Brinton (2007)
		DENV	Inhibition	3′-end viral genome captures TIA-1/TIAR	Li *et al*. (2002), Emara and Brinton (2007), Ward *et al*. (2011)
				3′-UTR interacts with G3BP1, G3BP2, Caprin1 and USP10	
		JEV	Inhibition	Recruits G3BP and USP10 to the perinuclear region	Tu *et al*. (2012)
				NS2A interact with PKR and prevent PKR dimerization	Ward *et al*. (2011)
		HCV	Induction	Activates PKR via the 5′- UTR of its genome	Toroney *et al*. (2010)
			Inhibition	NS5A protein binds to the PKR dimerization domain to inhibit PKR activation	Toroney *et al*. (2010)
				Modulate GADD34 and PP1 to de-phosphorylate eIF2α	Ruggieri *et al*. (2012)
		HCV-JFH1	Modulation	Redistributes several SG components to the HCV replication complex (RC)	Ariumi *et al*. (2011), Garaigorta *et al*. (2012), Pene *et al*. (2015)
		ZIKV	Inhibition	Induces the redistribution of TIAR to the viral RNA replication sites	Hou *et al*. (2017)
(−)ssRNA	*Arenaviridae*	JUNV	Inhibition	N and GPC impair the phosphorylation of eIF2α	Linero *et al*. (2011)
	*Bunyaviridae*	RVFV	Inhibition	RVFV attenuates mTOR signaling to inhibite 4EBP phosphorylation	Habjan *et al*. (2009), Ikegami *et al*. (2009), Hopkins *et al*. (2015)
	*Coronaviridae*	MERS-CoV	Inhibition	Accessory protein 4a bind viral dsRNA and prevent the viral dsRNA from PKR binding	Rabouw *et al*. (2016), Nakagawa *et al*. (2018)
	*Filoviridae*	EBOV	Inhibition	VP35 bind viral dsRNA and prevent the viral dsRNA from PKR binding	Nelson *et al*. (2016, Le Sage *et al*. (2017)
			Modulation	SG proteins are selectively sequestered within virus inclusions and co-localize with viral RNA to form inclusion-bound granules	Nelson *et al*. (2016)
	*Rhabdoviridae*	VSV	Modulation	Induces formation of the SG-like structures that co-localize with viral replication proteins and RNA	Dinh *et al*. (2013)
	*Paramyxoviridae*	MV	Inhibition	Encode a C protein to limit the accumulation of dsRNA	Okonski and Samuel (2013)
		SeV	Inhibition	Encode a C protein to limit the accumulation of dsRNA	Takeuchi *et al*. (2008)
				Trailer RNA captures TIAR from SG	Iseni *et al*. (2002)
		RSV	Induction	Mediated by PKR-dependent eIF2α phosphorylation.	Lindquist *et al*. (2011)
			Inhibition	Sequestration of OGT in IBs	Fricke *et al*. (2013)
		HPIV3	Inhibition	IBs shield viral RNAs from recognition by PKR	Hu *et al*. 2018)

Viruses also induce SG formation independent of eIF2α phosphorylation (Table 1). The most typical example is from Rift Valley fever virus (RVFV) (Habjan *et al.*^2009^; Ikegami *et al.*^2009^) (Fig. 2). RVFV (Hopkins *et al.*^2015^) infection attenuates Akt/mTOR signaling and inhibits 4EBP phosphorylation and translation of 5′-TOP mRNAs, subsequently leading to an inhibition of global protein translation. 5′-TOP–containing mRNAs are indeed targeted to PB, where RVFV uses these cellular mRNAs for cap-snatching (Hopkins *et al.*^2015^). This can reflect that SG may interact with PB in a process that is thought to result in the exchange of mRNA cargos (Kedersha *et al.*^2008^). Whether any virus induces SG formation to cause translation inhibition due to the destruction of eIF4G or eIF4A is worth exploring in the future.

### RNA Viruses Modulate SG Formation or Assembly

SG formation shuts off bulk host protein synthesis. However, all viruses depend on the host translation apparatus for their gene expression. Therefore, viruses, as intracellular parasites, have to modulate the stress response pathway and SG assembly to translate their proteins for virus replication. RNA viruses modulate stress response pathway at different levels of SG formation (Table 1): One is to regulate eIF2α phosphorylation, and the other is to regulate the process of SG nucleation.

#### RNA Viruses Modulate eIF2α Phosphorylation to Interfere with SG Formation

In some cases, viral gene products can act as antagonists by targeting the virus-activated eIF2α kinases such as PKR or even by directly modulating the phosphorylation of eIF2α (Fig. 3A). IAV NS1 (Khaperskyy *et al.*^2012^; Ng *et al.*^2013^), Middle East respiratory syndrome coronavirus (MERS-CoV) accessory protein 4a (Rabouw *et al.*^2016^; Nakagawa *et al.*^2018^), and Ebola virus (EBOV) multifunctional protein VP35 (Nelson *et al.*^2016^; Le Sage *et al.*^2017^) bind viral dsRNA and prevent the viral dsRNA from PKR binding to inhibit SG formation. Inhibition of SG formation facilitates the translation of viral mRNAs, leading to efficient virus replication. HCV NS5A protein (Toroney *et al.*^2010^) binds to the PKR dimerization domain to inhibit PKR activation. Japanese encephalitis virus (JEV) NS2A protein (Tu *et al.*^2012^) might similarly interact with PKR and then prevent PKR dimer formation. SeV (Takeuchi *et al.*^2008^) and measles virus (MV) (Okonski and Samuel 2013) encode a C protein to limit the accumulation of dsRNA to inhibit SG formation. It seems that a portion of RNA viruses encode RNA binding proteins to antagonize the activity of PKR. There are also other groups of RNA viruses which directly modulate the phosphorylation of eIF2α without PKR. Junín virus (JUNV) prevents SG assembly by impairing the phosphorylation of eIF2α through its nucleoprotein (N) and glycoprotein precursor (GPC) (Linero *et al.*^2011^). However, its mechanism remains to be elucidated, although it may be similar to HCV. Ruggieri and colleagues reported that HCV rapidly de-phosphorylated eIF2α through protein phosphatase 1 (PP1) and its regulatory subunit GADD34 (growth arrest and DNA-damage-inducible 34) (Kojima *et al.*^2003^; Clavarino *et al.*^2012^; Ruggieri *et al.*^2012^).

#### RNA Viruses Cleave/Sequester/Redistribute Stress Granule-Nucleating Proteins to Interfere with SG Assembly

Several RNA viruses have been shown to express viral effectors that can actively disrupt the accumulation of SG through cleavage of SG components (Fig. 3B). Poliovirus (PV) induces SG formation in early phase but induces SG disassembly at later stages via cleavage of G3BP by viral 3C, thus preventing SG formation (White *et al.*^2007^). Similar findings were also reported for encephalomyocarditis virus (EMCV) (Ng *et al.*^2013^), foot-and-mouth disease virus (FMDV) (Ye *et al.*^2018^; Visser *et al.*^2019^), coxsackievirus B3 (CBV3) (Fung *et al.*^2013^) and feline calicivirus (FCV) (Humoud *et al.*^2016^). FCV infection does not cause accumulation of SG, despite an increased phosphorylation of eIF2α (Humoud *et al.*^2016^). This is because FCV NS6Pro, a 3C-like proteinase, cleaves G3BP1 at a site different from the poliovirus 3C proteinase. Unlike FCV, murine norovirus (MNV) does not cleave G3BP1 and thus does not inhibit SG formation during virus infection (Humoud *et al.*^2016^). In general, picornaviruses inhibit SG formation by viral 2A/L or 3C cleaving the major components of SG. In recent study, Yang *et al*. found that the 2A protease of picornavirus (EV71, PV, CVA) inhibits typical SG formation, which is PKR and eIF2α phosphorylation-dependent, but induces atypical SG formation by cleaving eIF4GI to sequester cellular mRNA and release viral mRNA, thereby facilitating viral infection (Yang *et al.*^2018^). In other words, the 2A protease can transform the overall translation machinery favorable for productive viral infection by induction of atypical SG while blocking the typical SG in the presence of G3BP cleavage by viral 3C protease during viral infection (Yang *et al.*^2018^).

Redistribution or sequestering SG components to the viral replication sites is another strategy used by many viruses to impair SG assembly in infected cells (Fig. 3B). ZIKV infection induces the redistribution of TIAR to the viral RNA replication sites (Hou *et al.*^2017^); SeV Trailer RNA captures TIAR from SG (Iseni *et al.*^2002^); West Nile Virus (WNV) and Dengue virus (DENV) 3′-end viral genome captures TIA-1/TIAR (Li *et al.*^2002^; Emara and Brinton 2007; Xia *et al.*^2015^); DENV 3′-UTR interacts with G3BP1, G3BP2, Caprin1 and USP10 (Ward *et al.*^2011^; Reineke *et al.*^2015^); JEV recruits G3BP and USP10 to the perinuclear region through the interaction of JEV core protein with Caprin-1, a SG-associated cellular factor (Ward *et al.*^2011^). Theiler murine encephalomyelitis virus (TMEV) and mengovirus, a strain of EMCV, express the leader (L) protein to inhibit G3BP1 aggregation (Borghese and Michiels 2011). Sequestration or redistribution of SG components by viruses through protein–protein and protein-RNA interactions not only prevents SG assembly, but also facilitates viral genome replication. HCV-JFH1 infection redistributes several SG components, including G3BP1, ataxin-2 (ATX2), and poly(A)-binding protein 1 (PABP1), to the HCV replication complex (RC) (Ariumi *et al.*^2011^; Pene *et al.*^2015^), and co-opts G3BP1 to mediate efficient viral replication by interaction with NS5B and the 5′ end of the HCV minus-strand RNA (Ariumi *et al.*^2011^; Garaigorta *et al.*^2012^).

### RNA Virus Inclusion Bodies (IBs) Emerging as a New Strategy Used by Viruses to Resist SG

Studies on Human parainfluenza virus type 3 (HPIV3) (Hu *et al.*^2018^), RSV (Rincheval *et al.*^2017^), EBOV (Hoenen *et al.*^2012^), Rabies virus (RABV) (Lahaye *et al.*^2009^) and Vesicular stomatitis virus (VSV) (Heinrich *et al.*^2010^) showed that inclusion bodies (IBs) of negative stranded RNA viruses are the sites of viral RNA synthesis. A recent study suggested an emerging role of IBs in HPIV3 replication by shielding newly synthesized viral RNA from the antiviral effect of SG (Hu *et al.*^2018^) (Fig. 3B). Sequestration of O-linked N-acetylglucosamine (OGN) transferase (OGT), an enzyme that catalyzes the posttranslational addition of OGN to protein targets, in RSV IBs was also proposed to regulate SG nucleation and suppression of SG formation (Fricke *et al.*^2013^) (Fig. 3B). Viral transcription and replication of RABV take place within Negri bodies (NBs), which are IB-like structures (Lahaye *et al.*^2009^). RABV-induced SG are normally located closely to NBs. Viral mRNAs rather than viral genomic RNA accumulate in the SG-like structures together with cellular mRNAs were found to be specially transported from NBs to SG-like structures (Nikolic *et al.*^2016^). VSV infection also induces formation of the SG-like structures that co-localize with viral replication proteins and RNA, which are different from canonical SG (Dinh *et al.*^2013^). SG proteins (eIF4G, eIF3, PABP) are selectively sequestered within Ebola virus inclusion bodies and co-localize with viral RNA to form inclusion body-bound granules, which are functionally and structurally different from canonical SG, probably leading to inhibit the antiviral role of SG (Nelson *et al.*^2016^) (Fig. 3B). Collectively, these findings provoke more investigations on the roles of viral IBs in viral replication and resisting cellular responses.

**Fig. 4:**
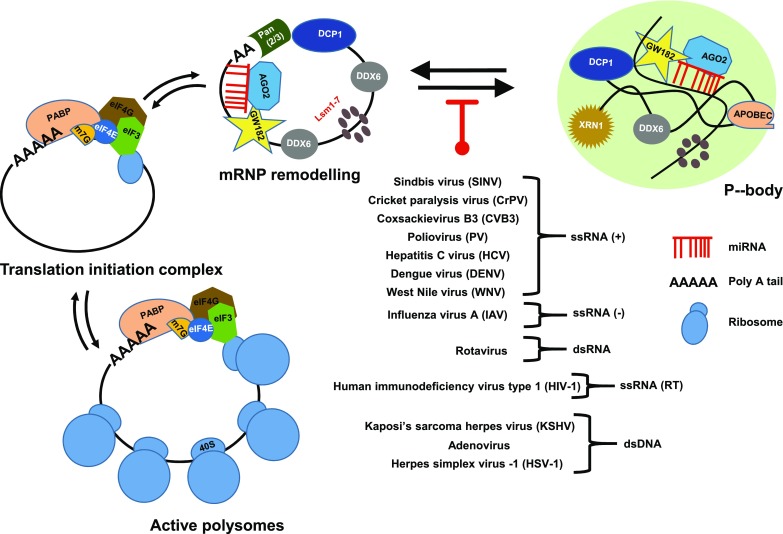
Disruption of PB assembly by viruses. The mRNA translation can be stopped for various reasons including the binding of miRNA. The translating mRNA can be stripped of ribosomes and the initiation complex can be collaps when binding to miRNA-RISC complex. The mRNPs targeted by PB components undergo three outcomes: 1. Translational inhibition; 2. Pan2/3-mediated deadenylation; 3. RNA decay by other associated RNA decay factors (e.g., Xrn1, Dcp1a, DDX6, and Lsm). Several RNA and DNA viruses which inhibit PB assembly are shown.

### DNA Viruses Regulate SG Formation

Unlike RNA viruses, the regulation of SG formation during infection with DNA viruses is poorly understood. It was reported that human cytomegalovirus (HCMV) infection modifies the unfolded protein response (UPR) and activates PERK (Fig. 2), but limiting the amount of phosphorylated eIF2α to maintain translation (Isler *et al.*^2005^). Kaposi’s sarcoma-associated herpesvirus (KSHV) ORF57 (Sharma *et al.*^2017^) interacts with PKR and PKR-activating protein (PACT) (Patel *et al.*^2000^) to inhibit PKR binding dsRNA and prevent PACT-PKR interaction in the PKR pathway (Li *et al.*^2006^), respectively. HCMV pTRS1 and pIRS1 antagonize PKR to facilitate virus replication (Ziehr *et al.*^2016^). The HSV-1 vhs (Sciortino *et al.*^2013^) and Us11 protein (Cassady and Gross 2002) play a key role in blocking the activation of PKR. Smiley and colleagues also demonstrated that infection with virion host shutoff protein (vhs)-defective herpes simplex virus 1 (HSV-1) triggers SG formation, and PKR is essential for SG formation in the absence of vhs (Dauber *et al.*^2016^) (Fig. 3A). Finnen *et al.* previously established that herpes simplex virus 2 (HSV-2) infection impacts stress granule accumulation in response to oxidative stress (Finnen *et al.*^2012^). They also demonstrated that disruption of SG is mediated by vhs (Finnen *et al.*^2014^), whose endoribonuclease activity is required to disrupt SG formation (Finnen *et al.*^2016^). HSV-2 vhs indeed have the ability to localize to SG (Finnen *et al.*^2016^) (Fig. 3B). This implies that removal of RNA from SG promotes its disassembly and that intact RNA is crucial for maintaining SG structure. It will be interesting to test the function of endoribonucleases in SG disassembly. Vaccinia virus (VV) sequesters crucial translation initiation factors, such as G3BP1, Caprin1, eIF4E, PABP and eIF4G (Katsafanas and Moss 2007; Simpson-Holley *et al.*^2011^; Zaborowska *et al.*^2012^), within cytoplasmic viral DNA factories to utilize SG components for different purposes (Fig. 3B). A recent study (Meng and Xiang 2019) suggested that the RNA granules are resulted from untranslated mRNA accumulation in viral DNA factories (Liu and Moss 2016) and TIA-1 is probably not required for granule formation and anti-poxviruses. Instead, the granules formation is most likely driven by an array of RNA–protein interactions and requires no specific SG components (Sivan *et al.*^2018^; Meng and Xiang 2019).

## Viral Regulation of RNA Processing Body Assembly

### Assembly of P-Bodies (PB)

PB were first reported in the scientific literature by Bashkirov *et al.* in 1997, and described as “small granules or discrete, prominent foci” or as the cytoplasmic location of the mouse exoribonuclease mXrn1p (Bashkirov *et al.*^1997^). Like SG, PB lack outer lipid membrane and now are recognized to be the sites where non-translating mRNAs accumulate for different fates including decay, storage, or returning to translation. A variety of enzymes involved in mRNA deadenylation (Ccr1, Caf1, Not1) (Sheth and Parker 2006), decapping (Dcp1/2, Lsm1-7, Edc3proteins) (Ingelfinger *et al.*^2002^; Yu *et al.*^2005^), nonsense-mediated decay (NMD) proteins (SMG5-6-7, UPF1) (Ingelfinger *et al.*^2002^; Durand *et al.*^2007^), in addition to scaffolding proteins (Ge-1/Hedls) (Yu *et al.*^2005^) and translation control factors (CPEB, eIF4E-T) (Andrei *et al.*^2005^; Wilczynska *et al.*^2005^), are the components of PB and used as routine markers to distinguish these granules. Nonetheless, some components (APOBEC3G, BRF1, DDX3, FAST, TTP, Rap55) (McEwen *et al.*^2005^; Sen and Blau 2005; Gallois-Montbrun *et al.*^2007^; Chen *et al.*^2008^) have also been shown to be shared by both SG and PB, suggesting a substantial linkage of these two structures and movement of mRNAs between both RNA granules. Interestingly, among these components, PB also include RNA-induced silencing complex (RISC) or miRNA associated argonaute (Ago) proteins (also shared with SG) and the GW182 protein which provides scaffolding activities for RISC to function, suggesting PB being the sites of miRNA mediated translation repression. The scaffolding activity of GW182 is critical for PB and knockdown of GW182 expression disrupts PB formation (Liu *et al.*^2005^). Notably, GW182 has been shown to bind to Ago2 which is critical for miRNA function and PB formation (Liu *et al.*^2005^). Recent evidence indicates that GW182 can recruit up to three molecules of Ago2 via its three GW motifs (glycine-tryptophan repeats) while each Ago protein has a single GW182-binding site (Elkayam *et al.*^2017^) (Fig. 4).

By applying fluorescence-activated particle sorting to purify PB in combination with mass spectrometry, Hubstenberger *et al*. identified 125 proteins that are significantly associated with PB (Hubstenberger *et al.*^2017^). By labeling several PB-localized proteins with a BirA (*E. coli* biotin ligase) enzyme in combination with mass spectrometry after streptavidin pulldown, Youn *et al*. identified 38 proteins in the PB (Youn *et al.*^2018^). ISGs (interferon stimulated genes) can also be found in PB during virus infection (Hebner *et al.*^2006^).

### RNA Viruses and PB

In comparison to viral regulation of SG, interaction of virus and PB was not much explored. It is an assumption that RNA viruses must regulate RNA decay processes/machinery to prevent degradation of virus genomes and mRNAs. Recently, some progress has been made to understand the relationship between PB components and some viruses in the context of viral gene expression. The data in published literatures are summarized in (Table 2). Mutation induced in the PB core components to affect the viral life cycles are well studied and tabulated in an earlier review (Beckham *et al.*^2007^). The report linking the assembly of yeast Ty3 retrotransposons virus—like particles with PB presented the first link between human retrovirus and PB (Checkley *et al.*^2010^). The later study revealed PB to be the site of anti-viral host factors APOBEC3G and APOBEC3F (A3G or A3F, apolipoprotein B mRNA-editing enzyme catalytic polypeptide 1-like) family of cytidine de-aminases, presumably representing a component of innate immunity against HIV (Wichroski *et al.*^2006^; Gallois-Montbrun *et al.*^2007^). In a different study, A3F was found to specifically interact with cellular signal recognition particle RNA (7SL RNA). Efficient packaging of 7SL RNA and A3F into HIV virons was mediated by the RNA-binding nucleocapsid domain of HIV-1 Gag (Wang *et al.*^2007^).

**Table 2:** Regulation of PB assembly by viruses

Genome	Virus family	Virus	P-bodies: accumulation/inhibition	Mechanism	References
dsDNA	*Adenoviridae*	Adenovirus	Inhibition	Redistribution of PB components by E4 11K including (Rck/p54/DDX6, Ago2, xrn1, Ge1, and Lsm-1)	Greer *et al*. (2011)
	*Herpesviridae*	Kaposi’s sarcoma herpes virus (KSHV)	Inhibition	Disruption of Ago2-GW182 interaction during lytic infection via ORF57	Sharma N. *et al*. unpublished
		Herpes simplex virus-1 (HSV-1)	Inhibition	Via ICP27	Sharma N. *et al*. unpublished
		Cytomegalovirus (HCMV)	Accumulation	Increased expression of Dcp1a, EDC4, Rck/p54/DDX6 and Rap55 proteins	Seto *et al*. (2014)
dsRNA	*Reoviridae*	Rotavirus	Inhibition	Sponge for RNA binding proteins which can redistribute several components of PB including Ago2, GW182 and Dcp1	Oceguera *et al*. (2018)
				Decreased expression of Pan3 and relocalization of Xrn1 and Dcp1	Bhowmick *et al*. (2015)
(+)ssRNA	*Flaviviridae*	West Nile virus (WNV)	Inhibition	Redistribution of Lsm1, GW182, DDX6, DDX3 and Xrn1 to viral replication factories (RF)	Chahar *et al*. (2013)
		Dengue virus (DENV)	Inhibition	N/A	Emara and Brinton (2007)
		Yellow fever virus (YFV)	Accumulation	sfRNA stalls Xrn1 and co-localizes at PB	Silva *et al*. (2010)
		Hepatitis C virus (HCV)	Inhibition	Redistribution of DDX6, Lsm1, Xrn1, PATL1 and Ago2 to lipid droplets	Ariumi *et al*. (2011)
				Dcp2 does not localize to viral factories	Ariumi *et al*. (2011)
	*Picornaviridae*	Poliovirus (PV)	Inhibition	Degradation of Xrn1, Dcp1a and Pan3 but not of GW182, EDC3/EDC4	Dougherty *et al*. (2011)
				Viral Protease 2A blocks PB formation	
		Coxsackievirus B3 (CVB3)	Inhibition	Cleavage of Xrn1, Dcp1a and Pan3	Dougherty *et al*. (2011)
	*Dicistroviridae*	Cricket paralysis virus (CrPV)	Inhibition	Disrupts only GW182/Dcp1 aggregate, but not Ago1/Ago2	Khong and Jan (2011)
	*Togaviridae*	Sindbis virus (SINV)	Inhibition	HuR-translocation out of the nucleus	Sokoloski *et al*. (2010)
(−)ssRNA	*Orthomyxoviridae*	Influenza virus A (IAV)	Inhibition	Interaction of RAP55 and NSP1	Mok *et al*. (2012)
	*Bunyaviridae*	Hantavirus	Accumulation	Cap snatching occurs in PB	Mir *et al*. (2008)
ssRNA-RT	*Retroviridae*	Human immunodeficiency virus type 1 (HIV-1)	Inhibition	HIV-1 mRNA interacts with DDX6, Ago 2 and APOBE3G and displaces from the PB	Nathans *et al*. (2009)
				Redistribution of PB components during the HIV-1 infection	Abrahamyan *et al*. (2010)
				Assembly intermediates (AIs) recruits DDX6 and ABCE1	Reed *et al*. (2012)
				miR-29a-HIV-1 mRNA interactions enhance viral mRNA association with RISC and PB	Nathans *et al*. (2009)
				MOV10 overexpression inhibits HIV-1 replication	Burdick *et al*. (2010), Furtak *et al*. (2010)

The bona fide and unique dependence of viruses on PB came from the studies on plant brome mosaic virus (BMV) (Beckham *et al.*^2007^). This study suggested the accumulation of BMV mRNAs in PB was an important step in RNA replication complex assembly for BMV, and possibly for other positive-strand RNA viruses. Nonetheless, many RNA viruses initiate the process of transcription of viral RNA by the process of ‘cap snatching’ which involves the acquisition of capped 5′ oligonucleotides from cellular mRNAs. Interestingly, PB were shown to serve as a pool of primers in the case of Hantavirus while its nucleocapsid protein, which accumulates in PB, binds 5′ caps with high affinity (Mir *et al.*^2008^).

The base-pair complementarity between a miRNA and a target mRNA dictates the miRNA to specifically repress posttranscriptional expression of mRNAs. Subsequent events in this process involve relocation of RNA-induced silencing complexes (RISCs) together with several other RNA binding proteins to form PB. In this context, HIV-1 mRNA interacts with RISC proteins and disrupting PB structures enhances viral production and infectivity, suggesting a role of PB against viral infection (Nathans *et al.*^2009^). Specific miR-29a-HIV-1 mRNA interaction was found to enhance viral mRNA association with RISC and PB proteins and regulate HIV-1 production and infectivity. HIV Nef interacts with Ago2 via its glycine-tryptophan region and functions as a viral suppressor of RNAi (Aqil *et al.*^2013^). While overexpression of Mov10, a component of PB and an ATP-dependent 5′-3′ RNA helicase, inhibits HIV production (Burdick *et al.*^2010^; Furtak *et al.*^2010^), Mov10 and APOBEC3G localization to PB is not required for HIV virion incorporation and antiviral activity (Izumi *et al.*^2013^). It becomes clear that Mov10 inhibits virus infection by enhancing RIG-I-MAVS-Independent IFN Induction (Cuevas *et al.*^2016^) and stabilizing A3G from degradation (Chen *et al.*^2017^).

The anticipated evidence of viral disruption of PB also came from the study with poliovirus (PV), a plus-strand RNA virus showing that PB are disrupted during PV infection in cells by 4 h post infection (Dougherty *et al.*^2011^). This function is attributed to viral proteinase 3C which degrades several components of PB including Xrn1 and Dcp1a, but not affecting others such as GW182, Edc3 and Edc4. Rotaviruses disassemble PB by using viral RNA as a sponge for RNA binding proteins to redistribute several PB components, including Ago2, GW182 and Dcp1 PB (Oceguera *et al.*^2018^). In fact, rotavirus disrupts PB through multiple mechanisms. The viral NSP1 protein seems to degrade PB component Pan3, while relocalizing other two components (Xrn1 and Dcp1a) (Bhowmick *et al.*^2015^). Intriguingly, exclusion of SG and PB components from the viroplasm is important for rotavirus replication and progeny virus production (Dhillon and Rao 2018).

### DNA Viruses and PB

While RNA viruses have evolved to co-opt or modulate the assembly of PB, this effect is rather unclear during infection by DNA viruses. Since most of the DNA viruses replicate and assemble in the nucleus, therefore as proposed for RNA viruses, accumulation of viral RNAs in PB for assembly cannot be a strategy required by DNA viruses. However, the close relationship of PB with translational repression reasonably provides a foundation for PB being antiviral cellular components against DNA viruses. Thus it is assumed that those factories suppressing mRNA translation would inhibit protein production of DNA viruses. To fight back, the DNA viruses have to develope strategies to bypass this antagonism mediated by PB for their survival and productive infection (Table 2).

Adenovirus E4 11 k, the product of E4 ORF3, accumulates viral late mRNA transcripts and at least five proteins of PB (Rck/p54/DDX6, Ago2, xrn1, Ge1, and Lsm-1) in the E4 11 k-induced cytoplasmic aggresomes. Redistribution of the PB components to the aggresomes, not to the PB, leads to inactivate or destroy these proteins. E4 11 k protein interacts with RNA helicase DDX6, one of the PB proteins, for its redistribution. Because PB are the sites for mRNA degradation, their alteration by E4 11 k suggests a role of E4 11 k in viral late mRNA accumulation (Greer *et al.*^2011^).

The role of PB in regulation of cytomegalovirus infection remains elusive. First, HCMV infection does not affect, but rather accumulates the formation of PB; second, PB formed during HCMV infection do not contain Ago2; third, HCMV prevents viral IE1 mRNA, a major IE gene product to encode a critical protein for viral gene expression and replication, from colocalization with PB (Seto *et al.*^2014^).

By generating a transgenic mice deficient of PB component LSm14A (or Rap55), recent studies showed that LSm14A plays a critical and specific role in the induction of antiviral cytokines (IFN-β, IFN-α, and IL-6) in dendritic cells (DCs). DNA viruses (HSV-1 and murine herpesvirus 68) and RNA virus VSV trigger this induction, but Sendai virus lacks such an effect (Anderson and Kedersha 2009; Liu *et al.*^2016^). LSm14A deficiency specifically downregulates MITA/STING (stimulator of interferon genes) level in DCs by impairing its nuclear mRNA precursor processing. In contrast to its role in mRNA decay, this study revealed a role of LSm14 in nuclear mRNA precursor processing and cell-specific regulatory mechanism of antiviral immune responses (Liu *et al.*^2016^).

KSHV kaposin B, a latent protein linked with cancer progression, induces PB dispersion (Corcoran *et al.*^2015^). Kaposin B activates the stress-responsive kinase MK2 in endothelial cells (ECs) to selectively block the decay of AU-rich mRNAs (ARE-mRNAs) which encode pro-inflammatory cytokines and angiogenic factors and to reprogram ECs through post-transcriptional control of EC gene expression and secretion. KSHV ORF57 protein inhibits the formation of PB during lytic infection by disrupting the essential interaction of Ago2 with GW182 (unpublished data). These data provide the first evidence that a tumor virus RNA-binding protein ORF57 antagonizes the RNA regulatory pathway of host antiviral defenses during lytic infection.

## Remarks and Perspectives

SG are highly dynamic structures (Jain *et al.*^2016^), which constantly exchange their components to regulate gene expression and are thought to be antiviral. SG composition appears to vary according to the inducing stimulus (Table 3). It’s clear that SG assembly/disassembly is a tightly regulated process which accompanies rearrangements of RNA and proteins (Wheeler *et al.*^2016^). Although significant advances have been made to understand how viruses regulate SG formation, our current knowledge is not suffucient to fully elucidate the machanism how SG are regulated in living cells. Further works are needed to address the following questions: First, is there any pathway to be a target for antiviral drug development? Second, do SG function as platforms that potentiate virus recognition? Third, is any unexplored pathway leading to SG formation which could be visualized by fluorescence in situ hybridization techniques—including single molecule RNA tracking methods in combination with super-resolution microscopy? Using viruses as a research tool will definitely teach us how the host fights virus infections and how the viruses get away from its host resistance.

**Table 3:** Viruses and SG components.

Genome	Virus family	Virus	SG components		Effects on viral replication*	References
			Proteins	RNAs		
dsDNA	*Poxviridae*	Vaccinia virus	G3BP, Caprin-1, eIF4G, eIF4E	Viral but not host mRNA	SG stimulate viral translation	Katsafanas and Moss (2007)
(+)ssRNA	*Picornaviridae*	EV71	Sam68, TIA-1, TIAR	Cellular but not viral mRNA	Induced aSG beneficial to viral translation	Yang *et al*. (2018)
		EV71-2A^C110S^	eIF4G, G3BP, TIA-1	Viral and cellular mRNA	EV71-2A^C110S^ induced tSG inhibit viral translation	Yang *et al*. (2018)
		TMEV L^M60V^	eIF3, TIA-1, PTB, G3BP	No viral RNA sequestered in SG	N/A	Borghese and Michiels (2011)
(−)ssRNA	*Rhabdoviridae*	Rabies Virus	G3BP1, TIA-1, PAPB	Viral and cellular mRNA	Efficient for virus infection	Nikolic *et al*. (2016)
		VSV	Viral replication proteins and TIA-1, TIAR, PCBP2	Viral RNA	No effect on viral protein synthesis despite eIF2 phosphorylation	Dinh *et al*. (2013)
	*Paramyxoviridae*	HPIV3	TIA-1, G3BP, eIF4A, eIF4E, eIF4G	+vRNA (the mRNA and the anti-genome RNA)	Inhibition of SG formation facilitates HPIV3 replication	Hu *et al*. (2018)
		RSV	G3BP, HuR, eIF3η, TIA-1	Genomic RNA	SG promote RSV replication	Lindquist *et al*. (2010)

*N/*A* not available; aSG, atypical SG; tSG, typical SG.

PB affect viral infections in multiple ways. Thus, it is difficult to generalize a common viral strategy in a particular virus group to interact with the components of PB. The noticed evidence is that viruses in the same family may show extremely distant behavior when they come to interact with PB (Table 2). More studies on virus interactions with PB will be required to characterize the PB to be proviral or antiviral in a context-dependent manner. Other key questions in the field for future studies are: (1) to understand the mechanisms that regulate PB formation in cells. Viral manipulation of PB may provide a better platform to understand this regulation; (2) to determine which viral RNA species preferentially travel through these RNA granules and which ones do not? (3) to identify the RNA elements dictating viral RNA to escape from SG and PB. Thus, discovery of virus regulations of PB assembly represents a new paradigm of virus-host interactions.

## References

[CR1] Abrahamyan LG, Chatel-Chaix L, Ajamian L, Milev MP, Monette A, Clement JF, Song R, Lehmann M, DesGroseillers L, Laughrea M, Boccaccio G, Mouland AJ (2010). Novel Staufen1 ribonucleoproteins prevent formation of stress granules but favour encapsidation of HIV-1 genomic RNA. J Cell Sci.

[CR2] Anderson P, Kedersha N (2008). Stress granules: the Tao of RNA triage. Trends Biochem Sci.

[CR3] Anderson P, Kedersha N (2009). RNA granules: post-transcriptional and epigenetic modulators of gene expression. Nat Rev Mol Cell Biol.

[CR4] Anderson P, Kedersha N, Ivanov P (2015). Stress granules, P-bodies and cancer. Biochim Biophys Acta.

[CR5] Andrei MA, Ingelfinger D, Heintzmann R, Achsel T, Rivera-Pomar R, Luhrmann R (2005). A role for eIF4E and eIF4E-transporter in targeting mRNPs to mammalian processing bodies. RNA.

[CR6] Aqil M, Naqvi AR, Bano AS, Jameel S (2013). The HIV-1 Nef protein binds argonaute-2 and functions as a viral suppressor of RNA interference. PLoS ONE.

[CR7] Ariumi Y, Kuroki M, Kushima Y, Osugi K, Hijikata M, Maki M, Ikeda M, Kato N (2011). Hepatitis C virus hijacks P-body and stress granule components around lipid droplets. J Virol.

[CR8] Bashkirov VI, Scherthan H, Solinger JA, Buerstedde JM, Heyer WD (1997). A mouse cytoplasmic exoribonuclease (mXRN1p) with preference for G4 tetraplex substrates. J Cell Biol.

[CR9] Beckham CJ, Light HR, Nissan TA, Ahlquist P, Parker R, Noueiry A (2007). Interactions between brome mosaic virus RNAs and cytoplasmic processing bodies. J Virol.

[CR10] Berlanga JJ, Ventoso I, Harding HP, Deng J, Ron D, Sonenberg N, Carrasco L, de Haro C (2006). Antiviral effect of the mammalian translation initiation factor 2alpha kinase GCN2 against RNA viruses. EMBO J.

[CR11] Bhowmick R, Mukherjee A, Patra U, Chawla-Sarkar M (2015). Rotavirus disrupts cytoplasmic P bodies during infection. Virus Res.

[CR12] Bordeleau ME, Cencic R, Lindqvist L, Oberer M, Northcote P, Wagner G, Pelletier J (2006). RNA-mediated sequestration of the RNA helicase eIF4A by Pateamine A inhibits translation initiation. Chem Biol.

[CR13] Borghese F, Michiels T (2011). The leader protein of cardioviruses inhibits stress granule assembly. J Virol.

[CR14] Buchan JR, Parker R (2009). Eukaryotic stress granules: the ins and outs of translation. Mol Cell.

[CR15] Burdick R, Smith JL, Chaipan C, Friew Y, Chen J, Venkatachari NJ, Delviks-Frankenberry KA, Hu WS, Pathak VK (2010). P body-associated protein Mov10 inhibits HIV-1 replication at multiple stages. J Virol.

[CR16] Burgess HM, Richardson WA, Anderson RC, Salaun C, Graham SV, Gray NK (2011). Nuclear relocalisation of cytoplasmic poly(A)-binding proteins PABP1 and PABP4 in response to UV irradiation reveals mRNA-dependent export of metazoan PABPs. J Cell Sci.

[CR17] Cassady KA, Gross M (2002). The herpes simplex virus type 1 U(S)11 protein interacts with protein kinase R in infected cells and requires a 30-amino-acid sequence adjacent to a kinase substrate domain. J Virol.

[CR18] Chahar HS, Chen S, Manjunath N (2013). P-body components LSM1, GW182, DDX3, DDX6 and XRN1 are recruited to WNV replication sites and positively regulate viral replication. Virology.

[CR19] Chan SW, Egan PA (2005). Hepatitis C virus envelope proteins regulate CHOP via induction of the unfolded protein response. FASEB J.

[CR20] Checkley MA, Nagashima K, Lockett SJ, Nyswaner KM, Garfinkel DJ (2010). P-body components are required for Ty1 retrotransposition during assembly of retrotransposition-competent virus-like particles. Mol Cell Biol.

[CR21] Chen D, Wilkinson CR, Watt S, Penkett CJ, Toone WM, Jones N, Bahler J (2008). Multiple pathways differentially regulate global oxidative stress responses in fission yeast. Mol Biol Cell.

[CR22] Chen C, Ma X, Hu Q, Li X, Huang F, Zhang J, Pan T, Xia J, Liu C (2017). Moloney leukemia virus 10 (MOV10) inhibits the degradation of APOBEC3G through interference with the Vif-mediated ubiquitin-proteasome pathway. Retrovirology.

[CR23] Clavarino G, Claudio N, Dalet A, Terawaki S, Couderc T, Chasson L, Ceppi M, Schmidt EK, Wenger T, Lecuit M, Gatti E, Pierre P (2012). Protein phosphatase 1 subunit Ppp1r15a/GADD34 regulates cytokine production in polyinosinic:polycytidylic acid-stimulated dendritic cells. Proc Natl Acad Sci U S A.

[CR24] Corcoran JA, Johnston BP, McCormick C (2015). Viral activation of MK2-hsp27-p115RhoGEF-RhoA signaling axis causes cytoskeletal rearrangements, p-body disruption and ARE-mRNA stabilization. PLoS Pathog.

[CR25] Cuevas RA, Ghosh A, Wallerath C (2016). MOV10 provides antiviral activity against RNA viruses by enhancing RIG-I-MAVS-independent IFN induction. J Immunol.

[CR26] Dang Y, Kedersha N, Low WK, Romo D, Gorospe M, Kaufman R, Anderson P, Liu JO (2006). Eukaryotic initiation factor 2alpha-independent pathway of stress granule induction by the natural product pateamine A. J Biol Chem.

[CR27] Dauber B, Poon D, Dos Santos T, Duguay BA, Mehta N, Saffran HA, Smiley JR (2016). The herpes simplex virus virion host shutoff protein enhances translation of viral true late mrnas independently of suppressing protein kinase R and stress granule formation. J Virol.

[CR28] Decker CJ, Parker R (2012). P-bodies and stress granules: possible roles in the control of translation and mRNA degradation. Cold Spring Harb Perspect Biol.

[CR29] Deng J, Harding HP, Raught B, Gingras AC, Berlanga JJ, Scheuner D, Kaufman RJ, Ron D, Sonenberg N (2002). Activation of GCN2 in UV-irradiated cells inhibits translation. Curr Biol.

[CR30] Dhillon P, Rao CD (2018). Rotavirus induces formation of remodeled stress granules and P bodies and their sequestration in viroplasms to promote progeny virus production. J Virol.

[CR31] Dinh PX, Beura LK, Das PB, Panda D, Das A, Pattnaik AK (2013). Induction of stress granule-like structures in vesicular stomatitis virus-infected cells. J Virol.

[CR32] Dougherty JD, White JP, Lloyd RE (2011). Poliovirus-mediated disruption of cytoplasmic processing bodies. J Virol.

[CR33] Durand S, Cougot N, Mahuteau-Betzer F, Nguyen CH, Grierson DS, Bertrand E, Tazi J, Lejeune F (2007). Inhibition of nonsense-mediated mRNA decay (NMD) by a new chemical molecule reveals the dynamic of NMD factors in P-bodies. J Cell Biol.

[CR34] Elkayam E, Faehnle CR, Morales M, Sun J, Li H, Joshua-Tor L (2017). Multivalent recruitment of human argonaute by GW182. Mol Cell.

[CR35] Emara MM, Brinton MA (2007). Interaction of TIA-1/TIAR with West Nile and dengue virus products in infected cells interferes with stress granule formation and processing body assembly. Proc Natl Acad Sci U S A.

[CR36] Eulalio A, Behm-Ansmant I, Schweizer D, Izaurralde E (2007). P-body formation is a consequence, not the cause, of RNA-mediated gene silencing. Mol Cell Biol.

[CR37] Finnen RL, Pangka KR, Banfield BW (2012). Herpes simplex virus 2 infection impacts stress granule accumulation. J Virol.

[CR38] Finnen RL, Hay TJ, Dauber B, Smiley JR, Banfield BW (2014). The herpes simplex virus 2 virion-associated ribonuclease vhs interferes with stress granule formation. J Virol.

[CR39] Finnen RL, Zhu M, Li J, Romo D, Banfield BW (2016). Herpes simplex virus 2 virion host shutoff endoribonuclease activity is required to disrupt stress granule formation. J Virol.

[CR40] Fournier MJ, Coudert L, Mellaoui S, Adjibade P, Gareau C, Cote MF, Sonenberg N, Gaudreault RC, Mazroui R (2013). Inactivation of the mTORC1-eukaryotic translation initiation factor 4E pathway alters stress granule formation. Mol Cell Biol.

[CR41] Fricke J, Koo LY, Brown CR, Collins PL (2013). p38 and OGT sequestration into viral inclusion bodies in cells infected with human respiratory syncytial virus suppresses MK2 activities and stress granule assembly. J Virol.

[CR42] Frolova E, Gorchakov R, Garmashova N, Atasheva S, Vergara LA, Frolov I (2006). Formation of nsP3-specific protein complexes during Sindbis virus replication. J Virol.

[CR43] Fujimura K, Sasaki AT, Anderson P (2012). Selenite targets eIF4E-binding protein-1 to inhibit translation initiation and induce the assembly of non-canonical stress granules. Nucleic Acids Res.

[CR44] Fung G, Ng CS, Zhang J, Shi J, Wong J, Piesik P, Han L, Chu F, Jagdeo J, Jan E, Fujita T, Luo H (2013). Production of a dominant-negative fragment due to G3BP1 cleavage contributes to the disruption of mitochondria-associated protective stress granules during CVB3 infection. PLoS ONE.

[CR45] Furtak V, Mulky A, Rawlings SA, Kozhaya L, Lee K, Kewalramani VN, Unutmaz D (2010). Perturbation of the P-body component Mov10 inhibits HIV-1 infectivity. PLoS ONE.

[CR46] Gallois-Montbrun S, Kramer B, Swanson CM, Byers H, Lynham S, Ward M, Malim MH (2007). Antiviral protein APOBEC3G localizes to ribonucleoprotein complexes found in P bodies and stress granules. J Virol.

[CR47] Garaigorta U, Heim MH, Boyd B, Wieland S, Chisari FV (2012). Hepatitis C virus (HCV) induces formation of stress granules whose proteins regulate HCV RNA replication and virus assembly and egress. J Virol.

[CR48] Garcia MA, Meurs EF, Esteban M (2007). The dsRNA protein kinase PKR: virus and cell control. Biochimie.

[CR49] Gilks N, Kedersha N, Ayodele M, Shen L, Stoecklin G, Dember LM, Anderson P (2004). Stress granule assembly is mediated by prion-like aggregation of TIA-1. Mol Biol Cell.

[CR50] Gorchakov R, Garmashova N, Frolova E, Frolov I (2008). Different types of nsP3-containing protein complexes in Sindbis virus-infected cells. J Virol.

[CR51] Greer AE, Hearing P, Ketner G (2011). The adenovirus E4 11 k protein binds and relocalizes the cytoplasmic P-body component Ddx6 to aggresomes. Virology.

[CR52] Habjan M, Pichlmair A, Elliott RM, Overby AK, Glatter T, Gstaiger M, Superti-Furga G, Unger H, Weber F (2009). NSs protein of rift valley fever virus induces the specific degradation of the double-stranded RNA-dependent protein kinase. J Virol.

[CR53] Harding HP, Novoa I, Zhang Y, Zeng H, Wek R, Schapira M, Ron D (2000). Regulated translation initiation controls stress-induced gene expression in mammalian cells. Mol Cell.

[CR54] Harding HP, Zhang Y, Bertolotti A, Zeng H, Ron D (2000). Perk is essential for translational regulation and cell survival during the unfolded protein response. Mol Cell.

[CR55] Hebner CM, Wilson R, Rader J, Bidder M, Laimins LA (2006). Human papillomaviruses target the double-stranded RNA protein kinase pathway. J Gen Virol.

[CR56] Heinicke LA, Wong CJ, Lary J, Nallagatla SR, Diegelman-Parente A, Zheng X, Cole JL, Bevilacqua PC (2009). RNA dimerization promotes PKR dimerization and activation. J Mol Biol.

[CR57] Heinrich BS, Cureton DK, Rahmeh AA, Whelan SP (2010). Protein expression redirects vesicular stomatitis virus RNA synthesis to cytoplasmic inclusions. PLoS Pathog.

[CR58] Hoenen T, Shabman RS, Groseth A, Herwig A, Weber M, Schudt G, Dolnik O, Basler CF, Becker S, Feldmann H (2012). Inclusion bodies are a site of ebolavirus replication. J Virol.

[CR59] Hopkins KC, Tartell MA, Herrmann C, Hackett BA, Taschuk F, Panda D, Menghani SV, Sabin LR, Cherry S (2015). Virus-induced translational arrest through 4EBP1/2-dependent decay of 5′-TOP mRNAs restricts viral infection. Proc Natl Acad Sci U S A.

[CR60] Hou S, Kumar A, Xu Z, Airo AM, Stryapunina I, Wong CP, Branton W, Tchesnokov E, Gotte M, Power C, Hobman TC (2017). Zika virus hijacks stress granule proteins and modulates the host stress response. J Virol.

[CR61] Hu Z, Wang Y, Tang Q, Yang X, Qin Y, Chen M (2018). Inclusion bodies of human parainfluenza virus type 3 inhibit antiviral stress granule formation by shielding viral RNAs. PLoS Pathog.

[CR62] Hubstenberger A, Courel M, Benard M, Souquere S, Ernoult-Lange M, Chouaib R, Yi Z, Morlot JB, Munier A, Fradet M, Daunesse M, Bertrand E, Pierron G, Mozziconacci J, Kress M, Weil D (2017). P-Body purification reveals the condensation of repressed mRNA regulons. Mol Cell.

[CR63] Humoud MN, Doyle N, Royall E, Willcocks MM, Sorgeloos F, van Kuppeveld F, Roberts LO, Goodfellow IG, Langereis MA, Locker N (2016). Feline calicivirus infection disrupts assembly of cytoplasmic stress granules and induces G3BP1 cleavage. J Virol.

[CR64] Ikegami T, Narayanan K, Won S, Kamitani W, Peters CJ, Makino S (2009). Rift Valley fever virus NSs protein promotes post-transcriptional downregulation of protein kinase PKR and inhibits eIF2alpha phosphorylation. PLoS Pathog.

[CR65] Ingelfinger D, Arndt-Jovin DJ, Luhrmann R, Achsel T (2002). The human LSm1-7 proteins colocalize with the mRNA-degrading enzymes Dcp1/2 and Xrnl in distinct cytoplasmic foci. RNA.

[CR66] Iseni F, Garcin D, Nishio M, Kedersha N, Anderson P, Kolakofsky D (2002). Sendai virus trailer RNA binds TIAR, a cellular protein involved in virus-induced apoptosis. EMBO J.

[CR67] Isler JA, Skalet AH, Alwine JC (2005). Human cytomegalovirus infection activates and regulates the unfolded protein response. J Virol.

[CR68] Ivanov PA, Chudinova EM, Nadezhdina ES (2003). Disruption of microtubules inhibits cytoplasmic ribonucleoprotein stress granule formation. Exp Cell Res.

[CR69] Ivanov P, Kedersha N, Anderson P (2018). Stress granules and processing bodies in translational control. Cold Spring Harb Perspect Biol.

[CR70] Izumi T, Burdick R, Shigemi M, Plisov S, Hu WS, Pathak VK (2013). Mov10 and APOBEC3G localization to processing bodies is not required for virion incorporation and antiviral activity. J Virol.

[CR71] Jackson RJ, Hellen CU, Pestova TV (2010). The mechanism of eukaryotic translation initiation and principles of its regulation. Nat Rev Mol Cell Biol.

[CR72] Jain S, Wheeler JR, Walters RW, Agrawal A, Barsic A, Parker R (2016). ATPase-modulated stress granules contain a diverse proteome and substructure. Cell.

[CR73] Jayabalan AK, Sanchez A, Park RY, Yoon SP, Kang GY, Baek JH, Anderson P, Kee Y, Ohn T (2016). NEDDylation promotes stress granule assembly. Nat Commun.

[CR74] Katsafanas GC, Moss B (2007). Colocalization of transcription and translation within cytoplasmic poxvirus factories coordinates viral expression and subjugates host functions. Cell Host Microbe.

[CR75] Kedersha N, Anderson P (2002). Stress granules: sites of mRNA triage that regulate mRNA stability and translatability. Biochem Soc Trans.

[CR76] Kedersha NL, Gupta M, Li W, Miller I, Anderson P (1999). RNA-binding proteins TIA-1 and TIAR link the phosphorylation of eIF-2 alpha to the assembly of mammalian stress granules. J Cell Biol.

[CR77] Kedersha N, Cho MR, Li W, Yacono PW, Chen S, Gilks N, Golan DE, Anderson P (2000). Dynamic shuttling of TIA-1 accompanies the recruitment of mRNA to mammalian stress granules. J Cell Biol.

[CR78] Kedersha N, Stoecklin G, Ayodele M, Yacono P, Lykke-Andersen J, Fritzler MJ, Scheuner D, Kaufman RJ, Golan DE, Anderson P (2005). Stress granules and processing bodies are dynamically linked sites of mRNP remodeling. J Cell Biol.

[CR79] Kedersha N, Tisdale S, Hickman T, Anderson P (2008). Real-time and quantitative imaging of mammalian stress granules and processing bodies. Methods Enzymol.

[CR80] Kedersha N, Ivanov P, Anderson P (2013). Stress granules and cell signaling: more than just a passing phase?. Trends Biochem Sci.

[CR81] Kedersha N, Panas MD, Achorn CA, Lyons S, Tisdale S, Hickman T, Thomas M, Lieberman J, McInerney GM, Ivanov P, Anderson P (2016). G3BP-Caprin1-USP10 complexes mediate stress granule condensation and associate with 40S subunits. J Cell Biol.

[CR82] Khaperskyy DA, Hatchette TF, McCormick C (2012). Influenza A virus inhibits cytoplasmic stress granule formation. FASEB J.

[CR83] Khong A, Jan E (2011). Modulation of stress granules and P bodies during dicistrovirus infection. J Virol.

[CR84] Kojima E, Takeuchi A, Haneda M, Yagi A, Hasegawa T, Yamaki K, Takeda K, Akira S, Shimokata K, Isobe K (2003). The function of GADD34 is a recovery from a shutoff of protein synthesis induced by ER stress: elucidation by GADD34-deficient mice. FASEB J.

[CR85] Lahaye X, Vidy A, Pomier C, Obiang L, Harper F, Gaudin Y, Blondel D (2009). Functional characterization of Negri bodies (NBs) in rabies virus-infected cells: evidence that NBs are sites of viral transcription and replication. J Virol.

[CR86] Le Sage V, Cinti A, McCarthy S, Amorim R, Rao S, Daino GL, Tramontano E, Branch DR, Mouland AJ (2017). Ebola virus VP35 blocks stress granule assembly. Virology.

[CR87] Leung AK, Vyas S, Rood JE, Bhutkar A, Sharp PA, Chang P (2011). Poly(ADP-ribose) regulates stress responses and microRNA activity in the cytoplasm. Mol Cell.

[CR88] Li W, Li Y, Kedersha N, Anderson P, Emara M, Swiderek KM, Moreno GT, Brinton MA (2002). Cell proteins TIA-1 and TIAR interact with the 3′ stem-loop of the West Nile virus complementary minus-strand RNA and facilitate virus replication. J Virol.

[CR89] Li S, Peters GA, Ding K, Zhang X, Qin J, Sen GC (2006). Molecular basis for PKR activation by PACT or dsRNA. Proc Natl Acad Sci U S A.

[CR90] Lindquist ME, Lifland AW, Utley TJ, Santangelo PJ, Crowe JE (2010). Respiratory syncytial virus induces host RNA stress granules to facilitate viral replication. J Virol.

[CR91] Lindquist ME, Mainou BA, Dermody TS, Crowe JE (2011). Activation of protein kinase R is required for induction of stress granules by respiratory syncytial virus but dispensable for viral replication. Virology.

[CR92] Linero FN, Thomas MG, Boccaccio GL, Scolaro LA (2011). Junin virus infection impairs stress-granule formation in Vero cells treated with arsenite via inhibition of eIF2alpha phosphorylation. J Gen Virol.

[CR93] Liu R, Moss B (2016). Opposing roles of double-stranded RNA effector pathways and viral defense proteins revealed with CRISPR-Cas9 Knockout cell lines and vaccinia virus mutants. J Virol.

[CR94] Liu J, Valencia-Sanchez MA, Hannon GJ, Parker R (2005). MicroRNA-dependent localization of targeted mRNAs to mammalian P-bodies. Nat Cell Biol.

[CR95] Liu TT, Yang Q, Li M, Zhong B, Ran Y, Liu LL, Yang Y, Wang YY, Shu HB (2016). LSm14A plays a critical role in antiviral immune responses by regulating MITA level in a cell-specific manner. J Immunol.

[CR96] Ma S, Bhattacharjee RB, Bag J (2009). Expression of poly(A)-binding protein is upregulated during recovery from heat shock in HeLa cells. FEBS J.

[CR97] Matsuki H, Takahashi M, Higuchi M, Makokha GN, Oie M, Fujii M (2013). Both G3BP1 and G3BP2 contribute to stress granule formation. Genes Cells.

[CR98] Mazroui R, Sukarieh R, Bordeleau ME, Kaufman RJ, Northcote P, Tanaka J, Gallouzi I, Pelletier J (2006). Inhibition of ribosome recruitment induces stress granule formation independently of eukaryotic initiation factor 2alpha phosphorylation. Mol Biol Cell.

[CR99] McCormick C, Khaperskyy DA (2017). Translation inhibition and stress granules in the antiviral immune response. Nat Rev Immunol.

[CR100] McEwen E, Kedersha N, Song B, Scheuner D, Gilks N, Han A, Chen JJ, Anderson P, Kaufman RJ (2005). Heme-regulated inhibitor kinase-mediated phosphorylation of eukaryotic translation initiation factor 2 inhibits translation, induces stress granule formation, and mediates survival upon arsenite exposure. J Biol Chem.

[CR101] McInerney GM, Kedersha NL, Kaufman RJ, Anderson P, Liljestrom P (2005). Importance of eIF2alpha phosphorylation and stress granule assembly in alphavirus translation regulation. Mol Biol Cell.

[CR102] Meng X, Xiang Y (2019). RNA granules associated with SAMD9-mediated poxvirus restriction are similar to antiviral granules in composition but do not require TIA1 for poxvirus restriction. Virology.

[CR103] Mir MA, Duran WA, Hjelle BL, Ye C, Panganiban AT (2008). Storage of cellular 5′ mRNA caps in P bodies for viral cap-snatching. Proc Natl Acad Sci U S A.

[CR104] Mok BW, Song W, Wang P, Tai H, Chen Y, Zheng M, Wen X, Lau SY, Wu WL, Matsumoto K, Yuen KY, Chen H (2012). The NS1 protein of influenza A virus interacts with cellular processing bodies and stress granules through RNA-associated protein 55 (RAP55) during virus infection. J Virol.

[CR105] Montero H, Rojas M, Arias CF, Lopez S (2008). Rotavirus infection induces the phosphorylation of eIF2alpha but prevents the formation of stress granules. J Virol.

[CR106] Nakagawa K, Narayanan K, Wada M, Makino S (2018). Inhibition of stress granule formation by middle east respiratory syndrome coronavirus 4a accessory protein facilitates viral translation, leading to efficient virus replication. J Virol.

[CR107] Nallagatla SR, Hwang J, Toroney R, Zheng X, Cameron CE, Bevilacqua PC (2007). 5′-triphosphate-dependent activation of PKR by RNAs with short stem-loops. Science.

[CR108] Nathans R, Chu CY, Serquina AK, Lu CC, Cao H, Rana TM (2009). Cellular microRNA and P bodies modulate host-HIV-1 interactions. Mol Cell.

[CR109] Nelson EV, Schmidt KM, Deflube LR, Doganay S, Banadyga L, Olejnik J, Hume AJ, Ryabchikova E, Ebihara H, Kedersha N, Ha T, Muhlberger E (2016). Ebola virus does not induce stress granule formation during infection and sequesters stress granule proteins within viral inclusions. J Virol.

[CR110] Ng CS, Jogi M, Yoo JS, Onomoto K, Koike S, Iwasaki T, Yoneyama M, Kato H, Fujita T (2013). Encephalomyocarditis virus disrupts stress granules, the critical platform for triggering antiviral innate immune responses. J Virol.

[CR112] Nikolic J, Civas A, Lama Z (2016). Rabies virus infection induces the formation of stress granules closely connected to the viral factories. PLoS Pathog.

[CR113] Oceguera A, Peralta AV, Martinez-Delgado G, Arias CF, Lopez S (2018). Rotavirus RNAs sponge host cell RNA binding proteins and interfere with their subcellular localization. Virology.

[CR114] Okonski KM, Samuel CE (2013). Stress granule formation induced by measles virus is protein kinase PKR dependent and impaired by RNA adenosine deaminase ADAR1. J Virol.

[CR115] Onomoto K, Jogi M, Yoo JS, Narita R, Morimoto S, Takemura A, Sambhara S, Kawaguchi A, Osari S, Nagata K, Matsumiya T, Namiki H, Yoneyama M, Fujita T (2012). Critical role of an antiviral stress granule containing RIG-I and PKR in viral detection and innate immunity. PLoS ONE.

[CR116] Panas MD, Ivanov P, Anderson P (2016). Mechanistic insights into mammalian stress granule dynamics. J Cell Biol.

[CR117] Patel CV, Handy I, Goldsmith T, Patel RC (2000). PACT, a stress-modulated cellular activator of interferon-induced double-stranded RNA-activated protein kinase, PKR. J Biol Chem.

[CR118] Pene V, Li Q, Sodroski C, Hsu CS, Liang TJ (2015). Dynamic interaction of stress granules, DDX3X, and IKK-alpha mediates multiple functions in hepatitis C virus infection. J Virol.

[CR119] Poblete-Duran N, Prades-Perez Y, Vera-Otarola J, Soto-Rifo R, Valiente-Echeverria F (2016). Who regulates whom? an overview of RNA granules and viral infections. Viruses.

[CR120] Protter DSW, Parker R (2016). Principles and properties of stress granules. Trends Cell Biol.

[CR121] Rabouw HH, Langereis MA, Knaap RC, Dalebout TJ (2016). Middle east respiratory coronavirus accessory protein 4a inhibits PKR-mediated antiviral stress responses. PLoS Pathog.

[CR122] Reed JC, Molter B, Geary CD, McNevin J, McElrath J, Giri S, Klein KC, Lingappa JR (2012). HIV-1 Gag co-opts a cellular complex containing DDX6, a helicase that facilitates capsid assembly. J Cell Biol.

[CR123] Reineke LC, Kedersha N, Langereis MA, van Kuppeveld FJ, Lloyd RE (2015). Stress granules regulate double-stranded RNA-dependent protein kinase activation through a complex containing G3BP1 and Caprin1. MBio.

[CR124] Rincheval V, Lelek M, Gault E, Bouillier C, Sitterlin D, Blouquit-Laye S, Galloux M, Zimmer C, Eleouet JF, Rameix-Welti MA (2017). Functional organization of cytoplasmic inclusion bodies in cells infected by respiratory syncytial virus. Nat Commun.

[CR125] Rojas M, Arias CF, Lopez S (2010). Protein kinase R is responsible for the phosphorylation of eIF2alpha in rotavirus infection. J Virol.

[CR126] Rozelle DK, Filone CM, Kedersha N, Connor JH (2014). Activation of stress response pathways promotes formation of antiviral granules and restricts virus replication. Mol Cell Biol.

[CR127] Ruggieri A, Dazert E, Metz P, Hofmann S, Bergeest JP, Mazur J, Bankhead P, Hiet MS, Kallis S, Alvisi G, Samuel CE, Lohmann V, Kaderali L, Rohr K, Frese M, Stoecklin G, Bartenschlager R (2012). Dynamic oscillation of translation and stress granule formation mark the cellular response to virus infection. Cell Host Microbe.

[CR128] Sciortino MT, Parisi T, Siracusano G, Mastino A, Taddeo B, Roizman B (2013). The virion host shutoff RNase plays a key role in blocking the activation of protein kinase R in cells infected with herpes simplex virus 1. J Virol.

[CR129] Sen GL, Blau HM (2005). Argonaute 2/RISC resides in sites of mammalian mRNA decay known as cytoplasmic bodies. Nat Cell Biol.

[CR130] Seto E, Inoue T, Nakatani Y, Yamada M, Isomura H (2014). Processing bodies accumulate in human cytomegalovirus-infected cells and do not affect viral replication at high multiplicity of infection. Virology.

[CR131] Sharma NR, Majerciak V, Kruhlak MJ, Zheng ZM (2017). KSHV inhibits stress granule formation by viral ORF57 blocking PKR activation. PLoS Pathog.

[CR132] Sheth U, Parker R (2006). Targeting of aberrant mRNAs to cytoplasmic processing bodies. Cell.

[CR133] Silva PA, Pereira CF, Dalebout TJ, Spaan WJ, Bredenbeek PJ (2010). An RNA pseudoknot is required for production of yellow fever virus subgenomic RNA by the host nuclease XRN1. J Virol.

[CR134] Simpson-Holley M, Kedersha N, Dower K, Rubins KH, Anderson P, Hensley LE, Connor JH (2011). Formation of antiviral cytoplasmic granules during orthopoxvirus infection. J Virol.

[CR135] Sivan G, Glushakow-Smith SG, Katsafanas GC, Americo JL, Moss B (2018). Human host range restriction of the vaccinia virus C7/K1 double deletion mutant is mediated by an atypical mode of translation inhibition. J Virol.

[CR136] Smith RW, Gray NK (2010). Poly(A)-binding protein (PABP): a common viral target. Biochem J.

[CR137] Sokoloski KJ, Dickson AM, Chaskey EL, Garneau NL, Wilusz CJ, Wilusz J (2010). Sindbis virus usurps the cellular HuR protein to stabilize its transcripts and promote productive infections in mammalian and mosquito cells. Cell Host Microbe.

[CR138] Srivastava SP, Kumar KU, Kaufman RJ (1998). Phosphorylation of eukaryotic translation initiation factor 2 mediates apoptosis in response to activation of the double-stranded RNA-dependent protein kinase. J Biol Chem.

[CR139] Stoecklin G, Kedersha N (2013). Relationship of GW/P-bodies with stress granules. Adv Exp Med Biol.

[CR140] Stohr N, Lederer M, Reinke C, Meyer S, Hatzfeld M, Singer RH, Huttelmaier S (2006). ZBP1 regulates mRNA stability during cellular stress. J Cell Biol.

[CR141] Takeuchi K, Komatsu T, Kitagawa Y, Sada K, Gotoh B (2008). Sendai virus C protein plays a role in restricting PKR activation by limiting the generation of intracellular double-stranded RNA. J Virol.

[CR142] Thomas MG, Loschi M, Desbats MA, Boccaccio GL (2011). RNA granules: the good, the bad and the ugly. Cell Signal.

[CR143] Toroney R, Nallagatla SR, Boyer JA, Cameron CE, Bevilacqua PC (2010). Regulation of PKR by HCV IRES RNA: importance of domain II and NS5A. J Mol Biol.

[CR144] Tourriere H, Chebli K, Zekri L, Courselaud B, Blanchard JM, Bertrand E, Tazi J (2003). The RasGAP-associated endoribonuclease G3BP assembles stress granules. J Cell Biol.

[CR145] Tsai WC, Gayatri S, Reineke LC, Sbardella G, Bedford MT, Lloyd RE (2016). Arginine demethylation of G3BP1 promotes stress granule assembly. J Biol Chem.

[CR146] Tu YC, Yu CY, Liang JJ, Lin E, Liao CL, Lin YL (2012). Blocking double-stranded RNA-activated protein kinase PKR by Japanese encephalitis virus nonstructural protein 2A. J Virol.

[CR147] Visser LJ, Medina GN, Rabouw HH, de Groot RJ, Langereis MA, de Los T, van Kuppeveld FJM (2019). Foot-and-mouth disease virus leader protease cleaves G3BP1 and G3BP2 and inhibits stress granule formation. J Virol.

[CR148] von der Haar T, Gross JD, Wagner G, McCarthy JE (2004). The mRNA cap-binding protein eIF4E in post-transcriptional gene expression. Nat Struct Mol Biol.

[CR149] Wang T, Tian C, Zhang W, Luo K, Sarkis PT, Yu L, Liu B, Yu Y, Yu XF (2007). 7SL RNA mediates virion packaging of the antiviral cytidine deaminase APOBEC3G. J Virol.

[CR150] Ward AM, Bidet K, Yinglin A, Ler SG, Hogue K, Blackstock W, Gunaratne J, Garcia-Blanco MA (2011). Quantitative mass spectrometry of DENV-2 RNA-interacting proteins reveals that the DEAD-box RNA helicase DDX6 binds the DB1 and DB2 3′ UTR structures. RNA Biol.

[CR151] Wek SA, Zhu S, Wek RC (1995). The histidyl-tRNA synthetase-related sequence in the eIF-2 alpha protein kinase GCN2 interacts with tRNA and is required for activation in response to starvation for different amino acids. Mol Cell Biol.

[CR152] Wheeler JR, Matheny T, Jain S, Abrisch R, Parker R (2016). Distinct stages in stress granule assembly and disassembly. Elife.

[CR153] White JP, Lloyd RE (2012). Regulation of stress granules in virus systems. Trends Microbiol.

[CR154] White JP, Cardenas AM, Marissen WE, Lloyd RE (2007). Inhibition of cytoplasmic mRNA stress granule formation by a viral proteinase. Cell Host Microbe.

[CR155] Wichroski MJ, Robb GB, Rana TM (2006). Human retroviral host restriction factors APOBEC3G and APOBEC3F localize to mRNA processing bodies. PLoS Pathog.

[CR156] Wilczynska A, Aigueperse C, Kress M, Dautry F, Weil D (2005). The translational regulator CPEB1 provides a link between dcp1 bodies and stress granules. J Cell Sci.

[CR157] Willis KL, Langland JO, Shisler JL (2011). Viral double-stranded RNAs from vaccinia virus early or intermediate gene transcripts possess PKR activating function, resulting in NF-kappaB activation, when the K1 protein is absent or mutated. J Biol Chem.

[CR158] Xia J, Chen X, Xu F, Wang Y, Shi Y, Li Y, He J, Zhang P (2015). Dengue virus infection induces formation of G3BP1 granules in human lung epithelial cells. Arch Virol.

[CR159] Yang X, Hu Z, Fan S, Zhang Q, Zhong Y, Guo D, Qin Y, Chen M (2018). Picornavirus 2A protease regulates stress granule formation to facilitate viral translation. PLoS Pathog.

[CR160] Ye X, Pan T, Wang D, Fang L, Ma J, Zhu X, Shi Y, Zhang K, Zheng H, Chen H, Li K, Xiao S (2018). Foot-and-mouth disease virus counteracts on internal ribosome entry site suppression by G3BP1 and Inhibits G3BP1-mediated stress granule assembly via post-translational mechanisms. Front Immunol.

[CR161] Youn JY, Dunham WH, Hong SJ, Knight JDR, Bashkurov M, Chen GI, Bagci H, Rathod B, MacLeod G, Eng SWM, Angers S, Morris Q, Fabian M, Cote JF, Gingras AC (2018). High-density proximity mapping reveals the subcellular organization of mRNA-associated granules and bodies. Mol Cell.

[CR162] Yu JH, Yang WH, Gulick T, Bloch KD, Bloch DB (2005). Ge-1 is a central component of the mammalian cytoplasmic mRNA processing body. RNA.

[CR163] Zaborowska I, Kellner K, Henry M, Meleady P, Walsh D (2012). Recruitment of host translation initiation factor eIF4G by the vaccinia virus ssDNA-binding protein I3. Virology.

[CR164] Ziehr B, Vincent HA, Moorman NJ (2016). Human cytomegalovirus pTRS1 and pIRS1 antagonize protein kinase R To facilitate virus replication. J Virol.

[CR165] Zoncu R, Efeyan A, Sabatini DM (2011). mTOR: from growth signal integration to cancer, diabetes and ageing. Nat Rev Mol Cell Biol.

